# Prime-boost vaccination targeting prostatic acid phosphatase (PAP) in patients with metastatic castration-resistant prostate cancer (mCRPC) using Sipuleucel-T and a DNA vaccine

**DOI:** 10.1186/s40425-018-0333-y

**Published:** 2018-03-13

**Authors:** Ellen Wargowski, Laura E. Johnson, Jens C. Eickhoff, Lauren Delmastro, Mary Jane Staab, Glenn Liu, Douglas G. McNeel

**Affiliations:** 1 University of Wisconsin Carbone Cancer Center, 1111 Highland Avenue, Madison, WI 53705 USA; 2 Department of Biostatistics, University of Wisconsin, Madison, WI 53792 USA

**Keywords:** Sipuleucel-T, DNA vaccine, Prostate cancer, Prostatic acid phosphatase, Immune monitoring, Clinical trial

## Abstract

**Background:**

Prostatic acid phosphatase (PAP) is a prostate tumor antigen, and the target of the only FDA-approved anti-tumor vaccine, sipuleucel-T. We have previously reported in two clinical trials that a DNA vaccine encoding PAP (pTVG-HP) could elicit PAP-specific, Th1-biased T cells in patients with PSA-recurrent prostate cancer. In the current pilot trial we sought to evaluate whether this vaccine could augment PAP-specific immunity when used as a booster to immunization with sipuleucel-T in patients with metastatic, castration-resistant prostate cancer (mCRPC).

**Methods:**

Eigthteen patients with mCRPC were randomized to receive sipuleucel-T alone or followed by intradermal immunization with pTVG-HP DNA vaccine. Patients were followed for time to progression, and immune monitoring was conducted at defined intervals.

**Results:**

Overall, patients were followed for a median of 24 months. 11/18 patients completed treatments as per protocol. No treatment-associated events > grade 2 were observed. Th1-biased PAP-specific T-cell responses were detected in 11/18 individuals, and were not statistically different between study arms. Higher titer antibody responses to PAP were detectable in patients who received pTVG-HP booster immunizations. Median time to progression was less than 6 months and not statistically different between study arms. The median overall survival for all patients was 28 months.

**Conclusions:**

These findings suggest that prime-boost vaccination can augment and diversify the type of immunity elicited with anti-tumor vaccination in terms of T-cell and humoral immunity. Future studies will explore DNA as priming immunization rather than a booster immunization.

**Trial registration:**

NCT01706458.

## Background

Sipuleucel-T was approved by the U.S. Food and Drug Administration (FDA) for the treatment of patients with metastatic, castration-resistant prostate cancer based on data from a randomized clinical trial demonstrating an improvement in overall survival compared to placebo [1]. While the median improvement in overall survival was only 4 months, this is as comparable to other agents that have been approved for this stage of prostate cancer, including docetaxel [2, 3], cabazitaxel [4], abiraterone [5], radium-223 [6], and enzalutamide [7]. Subsequent retrospective studies have suggested that patients with lower burdens of disease, and those who developed evidence of immunity to the prostatic acid phosphatase (PAP) target antigen with either antigen-specific IgG or T cells, might have had a superior outcome in terms of longer overall survival [8, 9]. Consequently, these findings suggest that the target of this vaccine, PAP, is a rational vaccine target antigen for prostate cancer treatment. Moreover, these findings suggest that using combination vaccine approaches to increase the immunological activity of sipuleucel-T to PAP might lead to superior clinical outcomes.

We have evaluated PAP-targeted vaccines using plasmid DNA as the means of antigen delivery [10]. In two phase I trials evaluating dose and schedule in patients with non-metastatic prostate cancer (castration-sensitive and castration-resistant), we found vaccination to be safe and able to elicit PAP-specific CD4+ and CD8+ T cells with a Th1 phenotype [11, 12]. Unlike results from trials using sipuleucel-T, DNA vaccination did not elicit PAP-specific antibodies in either trial. The frequency of PAP-specific T cells was augmented with subsequent immunization, and the development of durable Th1-biased immune responses (detectable up to one year after treatment) appeared to be associated with favorable changes in PSA doubling time [12, 13]. Based on these results, a randomized phase II trial evaluating this vaccine is currently underway to determine whether treatment can delay the time to development of metastases in patients with biochemically recurrent prostate cancer (NCT01341652).

The ability of a DNA vaccine to elicit and augment Th1-biased immunity to PAP suggests it might be useful in a prime-boost strategy with sipuleucel-T, particularly since both vaccines target the same PAP antigen. Given that sipuleucel-T is an approved therapy delivered three times at two-week intervals, we sought to evaluate an approach in which DNA immunization was delivered after sipuleucel-T, as a booster immunization. We describe here the results of a pilot randomized clinical trial (NCT01706458) in which patients with mCRPC received sipuleucel-T alone (3 times at 2-week intervals), or sipuleucel-T (3 times at 2-week intervals) followed by DNA immunization 4 times at 2-week intervals, and then at months 6 and 9 after study initiation. The primary endpoint of the study was to determine if DNA vaccination could augment PAP-specific effector and memory T cells following treatment with sipuleucel-T. Secondary and exploratory objectives included effects on other measures of immunity, progression-free survival, and overall survival.

## Methods

### Investigational agent and regulatory information

pTVG-HP is a plasmid DNA encoding the full-length human PAP (ACPP gene) cDNA downstream of a eukaryotic promoter [14]. The study protocol was reviewed and approved by all local (University of Wisconsin Human Subjects’ Review Board), and federal (FDA, NIH Recombinant DNA Advisory Committee) entities. All patients gave written informed consent for participation.

### Patient population

Male patients with a histological diagnosis of prostate adenocarcinoma and PSA recurrence following castration (surgical or ongoing luteinizing hormone-releasing hormone agonist therapy) were eligible, provided they had evidence of metastatic disease by CT of abdomen/pelvis and/or bone scintigraphy. Progressive disease following the last treatment was required, as per Prostate Cancer Working Group 2 criteria [15], and patients were required to be at least 4 weeks from prior treatment. A minimum of three PSA values, obtained from the same clinical laboratory over at least a 12-week period of time prior to registration, was required to calculate a PSA doubling time. Patients were required to have an Eastern Cooperative Oncology Group performance score of ≤ 2, and normal bone marrow, liver and renal function as defined by a WBC ≥ 2000/μL, ANC ≥ 1000 / mm^3^, hemoglobin ≥ 9.0 g/dL, platelet count ≥ 100,000/μL, AST and ALT ≤ 2.5× institutional upper limit of normal, and serum creatinine < 2.0 mg/dL. Patients were excluded if they had symptomatic disease (defined as requiring opioid analgesics for the treatment of pain attributed to a metastatic lesion), or been treated with chemotherapy within 6 months, or radiation therapy or systemic corticosteroid therapy (≥ 1 mg dose equivalent prednisone daily) within 4 weeks, of registration. Patients were further excluded if they had a history of HIV, hepatitis B, or hepatitis C infection, or if they had received prior sipuleucel-T treatment.

### Study design and procedures

**Fig. 1:**
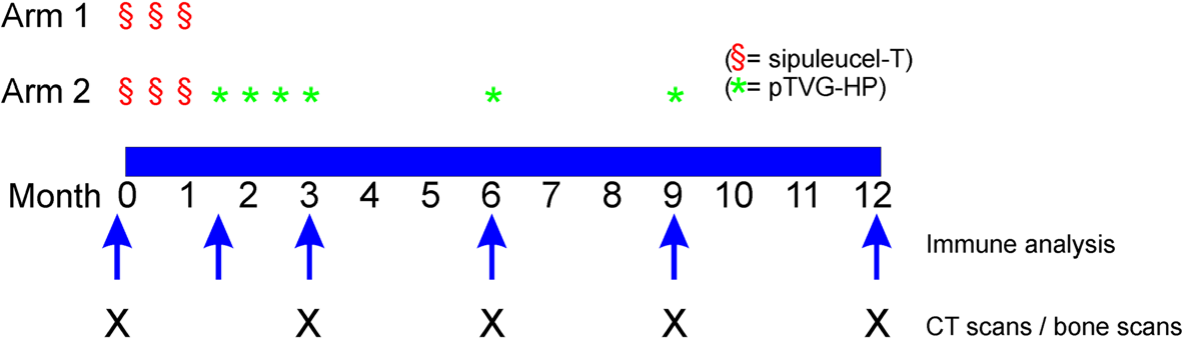
Trial Schema. Trial schema with schedules of vaccine administrations, immune analysis, and clinical staging evaluations, indicated

This study was an open-label, single institution, two-arm pilot trial (Fig. 1). The primary endpoint was to determine if booster immunizations could augment the antigen-specific T-cell immune response rate. The accrual goal was 28 patients to identify an increase in immune response rate by 50% with ≥ 80% power at a one-sided 5% significance level. Subjects in Arm 1 received sipuleucel-T as per standard of care, three times at two-week intervals. Subjects in Arm 2 received sipuleucel-T as per standard of care, three times at two-week intervals, and then received four biweekly immunizations with pTVG-HP (100 μg per immunization, co-administered intradermally with 200 μg GM-CSF adjuvant (sargramostim, Genzyme, Cambride, MA) at weeks 6, 8, 10, and 12, and then further immunizations at months 6 and 9. DNA immunizations were delivered as an intradermal injection in the deltoid region with a 28-guage needle and syringe. Patients underwent a leukapheresis procedure within two weeks prior to the first sipuleucel-T product collection and at month 6 for immunological monitoring. Additional 100 mL blood draws were performed at weeks 6, 12, and month 9 and 12 for immunological monitoring. All subjects were followed for at least one year, with staging CT scans of the abdomen and pelvis, and bone scintigraphy, performed every 12 weeks. Patients remained in long-term follow-up for up to five years to identify any potential long-term risks. Patients came off study at month 12, at the time of radiographic progression, at any time of undue toxicity, or at the discretion of the patient or treating physician that other therapies for prostate cancer were warranted. In order to account for possible delayed treatment effects, patients did not come off trial for radiographic progression at week 12 unless there was symptomatic progression requiring new therapy. Radiographic progression was defined using modified RECIST/PCWG2 criteria, however using scans obtained at week 12 as the baseline study for comparison. Patients also received a tetanus immunization immediately following the baseline leukapheresis. Blood tests were performed every 6–12 weeks and included CBC, creatinine, SGOT, alkaline phosphatase, amylase, LDH, bilirubin, serum PAP, and serum PSA. Serum testosterone was performed at baseline to confirm that patients were functionally castrate (testosterone levels < 50 ng/mL). All toxicities were graded according to the NCI Common Terminology Criteria Grading System, version 3.

**Table 1:** Demographics. Demographics for all patients enrolled

	Treatment Arm		
	1 (*n* = 9)	2 (*n* = 9)	Overall
Age (years)			
Median (range)	75 (67–82)	72 (66–85)	74 (66–85)
Race (n, %)			
White / Caucasian	9 (100%)	9 (100%)	18 (100%)
ECOG Performance Status (n, %)			
0	9 (100%)	8 (89%)	17 (94%)
1	0 (0%)	1 (11%)	1 (6%)
Gleason score (n, %)			
< 7	1 (11%)	2 (22%)	3 (17%)
7	3 (33%)	4 (44%)	7 (39%)
8	0 (0%)	2 (22%)	2 (11%)
≥ 9	5 (56%)	0 (0%)	5 (28%)
Unknown	0 (0%)	1 (11%)	1 (6%)
Metastatic sites (n, %)			
Visceral	2 (22%)	0 (0%)	2 (11%)
Bone	2 (22%)	5 (56%)	7 (39%)
Distant lymph nodes	6 (67%)	4 (44%)	10 (56%)
Baseline PSA (ng/mL)			
Median (range)	32.6 (4.17–1090)	11.2 (2.09–68.4)	16.25 (2.09–1090),
Baseline PSA doubling time (months)			
Median (range)	2.8 (1.4–63.9)	2.2 (1.0–25.8)	2.55 (1.0–63.)

### Immune analyses

For each time point, measures of antigen-specific T-cell immunity were performed with fresh (not cryopreserved) peripheral blood mononuclear cells (PBMC), and without in vitro stimulation prior to analysis. ELISPOT for IFNγ and granzyme B release were performed as previously described in 8-well replicates [12]. For these analyses, protein antigens (PAP (Fitzgerald Industries, Acton, MA), PSA (Fitzgerald), tetanus toxoid (EMD Millipore, Billerica, MA), and GM-CSF (Leukine®, Sanofi, Bridgewater, NJ)) and human AB serum (Valley Biomedical, Winchester, VA) used were from the same lots to control for possible variation over time. A response resulting from immunization was defined as a PAP-specific response detectable more than once post-treatment that was both significant (compared to media only control), at least 3-fold higher than the pre-treatment value, and with a frequency > 1:100,000 PBMC. For peptide-specific evaluation over time, cryopreserved PBMC were thawed, stimulated in vitro with 0.5 μg/mL of peptide for 7–11 days, washed, and then evaluated by ELISPOT as indicated above. IgG specific for PAP, PSA, GM-CSF or tetanus toxoid were evaluated by indirect ELISA, as previously described [16]. Peptide arrays (Roche-Nimblegen, Madison, WI) containing 16-mer peptides spanning the amino acid sequence of PAP, overlapping by 4 amino acids, were screened for IgG antibody responses, using sera diluted 1:100 from pre-treatment or 6-month blood collections, and assessed for mean fluorescence to each peptide, as previously reported [17].

### Clinical response evaluation

Staging studies (CT of abdomen/pelvis and bone scintigraphy) were performed every 12 weeks, or as clinically indicated. PSA values were collected from the same clinical laboratory at 6–12 week intervals. A minimum of three PSA values collected over a 12-week period of time, with PSA values up to 6 months, and including the screening value, was used to determine the pre-treatment PSA DT. All values collected on study up to 6 months were used to determine the post-treatment PSA DT. PSA DT was calculated as log(2) divided by the slope parameter estimate of the linear regression model of the log-transformed PSA values on time.

### Statistical analysis

Demographic characteristics and clinical outcomes were summarized in frequencies and percentages or medians and ranges. Immunological parameters were analyzed descriptively and displayed in graphic format using profile plots. Time to radiographic progression and overall survival were analyzed using the Kaplan-Meier method and compared between arms using the log-rank test. Statistical analyses were conducted using SAS software version 9.2 (SAS Institute Inc., Carey, NC).

## Results

### Patient population and course of study

Eithteen patients were enrolled in this trial between 2013 and 2016 at the University of Wisconsin Carbone Cancer Center. The median age of participants was 74 years (range 66–85 years), the median serum PSA at the time of study entry for all participants was 16.25 ng/mL (range 2.09–1090 ng/mL), and the median pre-treatment PSA DT for all patients was 2.55 months (range 1.0–63.9 months). Other demographics are shown in Table 1. Two patients in Arm 1 had visceral disease, which was slightly imbalanced relative to Arm 2. As shown in Table 2, few treatment associated adverse events were observed, and no events were greater than grade 2. Most events were grade 1 or grade 2 flu-like events of limited duration typical for vaccines, including chills, fatigue, fever, and headache. The majority of patients receiving the DNA vaccine experienced grade 1 injection site reactions. Progression was defined as radiographic progression after the first 3-month staging evaluation, and patients were not to be removed from study on the basis of PSA rise only. No patients came off trial due to toxicity. However, 6 patients (3 in each study arm) came off study prior to 6 months at physicians’ discretion for clinical progression requiring other therapies. All patients received all sipuleucel-T infusions, however one patient in Arm 2 (ID012) came off study for symptomatic deterioration prior to receiving any DNA vaccine. Patients were followed for 2 years following study completion, and the median length of follow-up for all patients was 24 months. The trial was closed before it reached its accrual goal of 28 patients due to slow accrual and that it appeared unlikely to meet its primary immunological endpoint.

**Table 2:** Adverse events. All adverse events at least possibly attributed to study treatment are shown. Numbers represent the number of patients per arm experience a particular event at any point during the treatment period, with the highest grade reported for any single individual. Adverse event grade is according to NCI CTCAE v.3

	Grade 1		Grade 2		Grade 3–5	
	Arm 1	Arm 2	Arm 1	Arm 2	Arm 1	Arm 2
General / Constitutional						
Chills	3 (33%)	1 (11%)		1 (11%)		
Fatigue	2 (22%)	2 (22%)		1 (11%)		
Fever	1 (11%)	1 (11%)				
Malaise		1 (11%)				
Pain		2 (22%)				
Headache		3 (33%)				
Injection site reaction		6 (67%)				
Gastrointestinal						
Nausea	3 (33%)	3 (33%)				
Diarrhea		1 (11%)				
Vascular						
Hypertension	1 (11%)					
Hot flashes		1 (11%)				
Hematologic						
Anemia		1 (11%)				
Metabolism						
Anorexia		1 (11%)				
Musculoskeletal						
Arthralgia		1 (11%)				
Back pain				1 (11%)		
Myalgia		1 (11%)				
Pain in extremity		1 (11%)				

### Immunological evaluation

**Fig. 2:**
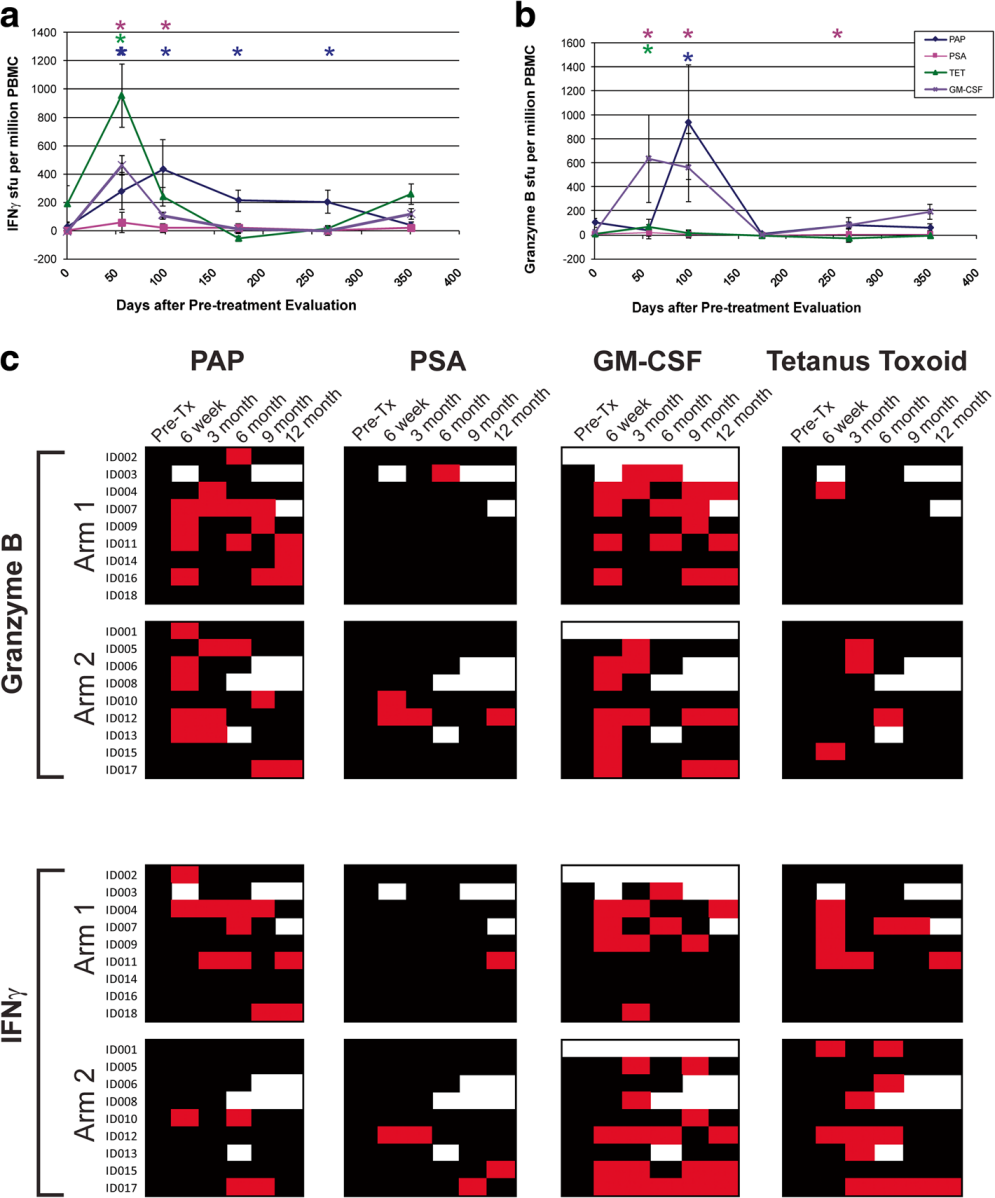
Sipuleucel-T and DNA immunization elicits PAP-specific cellular immune responses. **a** ELISPOT was used to determine the frequency of antigen-specific IFNγ-secreting T-cells over multiple time points. Shown are results for one patient (ID004) obtained in real-time at each time point. **b** ELISPOT was also used to determine the frequency of antigen-specific granzyme B-secreting T-cells over multiple time points for the same individual. Asterisks denote positive immune responses elicited, defined as an antigen-specific response (statistically higher than the media-only control, *p* < 0.05 by t-test) that was at least 3-fold higher than baseline and with a frequency > 1:100,000 cells. **c** Shown are the comprehensive summary data from immune monitoring by evaluation for PAP-, PSA-, GM-CSF, or tetanus-specific immune responses as assessed by IFNγ or granzyme B secretion by ELISPOT. A positive immune response (red) was defined as an antigen-specific response (statistically higher than the media-only control, *p* < 0.05 by t-test) that was at least 3-fold higher than baseline and with a frequency > 1:100,000 cells. Black squares indicate a time point where an assessment was performed, but in which immune response criteria were not met. White squares indicate time points where no data was collected

The primary immunological goal of this study was to determine whether booster immunizations with a DNA vaccine encoding PAP could augment the number of PAP-specific effector and memory T cells following treatment with sipuleucel-T, or prolong the duration of detectable T-cell response. All subjects received a tetanus booster immunization prior to beginning the immunization series, providing a separate test of an individual’s immune responsiveness [12]. Responses to PSA, a non-target prostate-specific protein, were concurrently evaluated, as were responses to GM-CSF, a component of the PA2024 fusion protein used in the preparation of sipuleucel-T. Samples were evaluated for antigen-specific IFNγ or granzyme B secretion by ELISPOT, and the detection of statistically significant antigen-specific responses, that were at least 3-fold over the baseline value, and detectable more than once post-treatment, were used to define immune response to a particular antigen, as previously reported [12]. An example of these analyses conducted over time for one individual treated with sipuleucel-T alone (Arm 1) is shown in Fig. 2. In this individual, PAP-specific, tetanus-specific, and GM-CSF-specific IFNγ (Fig. 2a) and/or granzyme B (Fig. 2b) was detected. A summary of IFNγ and granzyme B response for all patients is shown in Fig. 2c. As shown, 11/18 (61%) individuals developed PAP-specific IFNγ and/or granzyme B-secreting T-cell responses that were detectable at least twice in follow-up. Of these, 6 were in Arm 1, and 5 were in Arm 2 (with DNA vaccine), and hence a higher immune response rate was not detected in patients receiving both vaccines. 11/16 (69%) developed GM-CSF-specific IFNγ- and/or granzyme B-secreting T-cell responses that were detectable at least twice in follow up. Of note, 6/16 (38%) individuals developed persistent immunity to GM-CSF but not PAP. Responses to tetanus toxoid were predominantly IFNγ-biased, as we have previously demonstrated [12]. T-cell responses to PSA were rare (1/18, 5%) as expected.

**Fig. 3:**
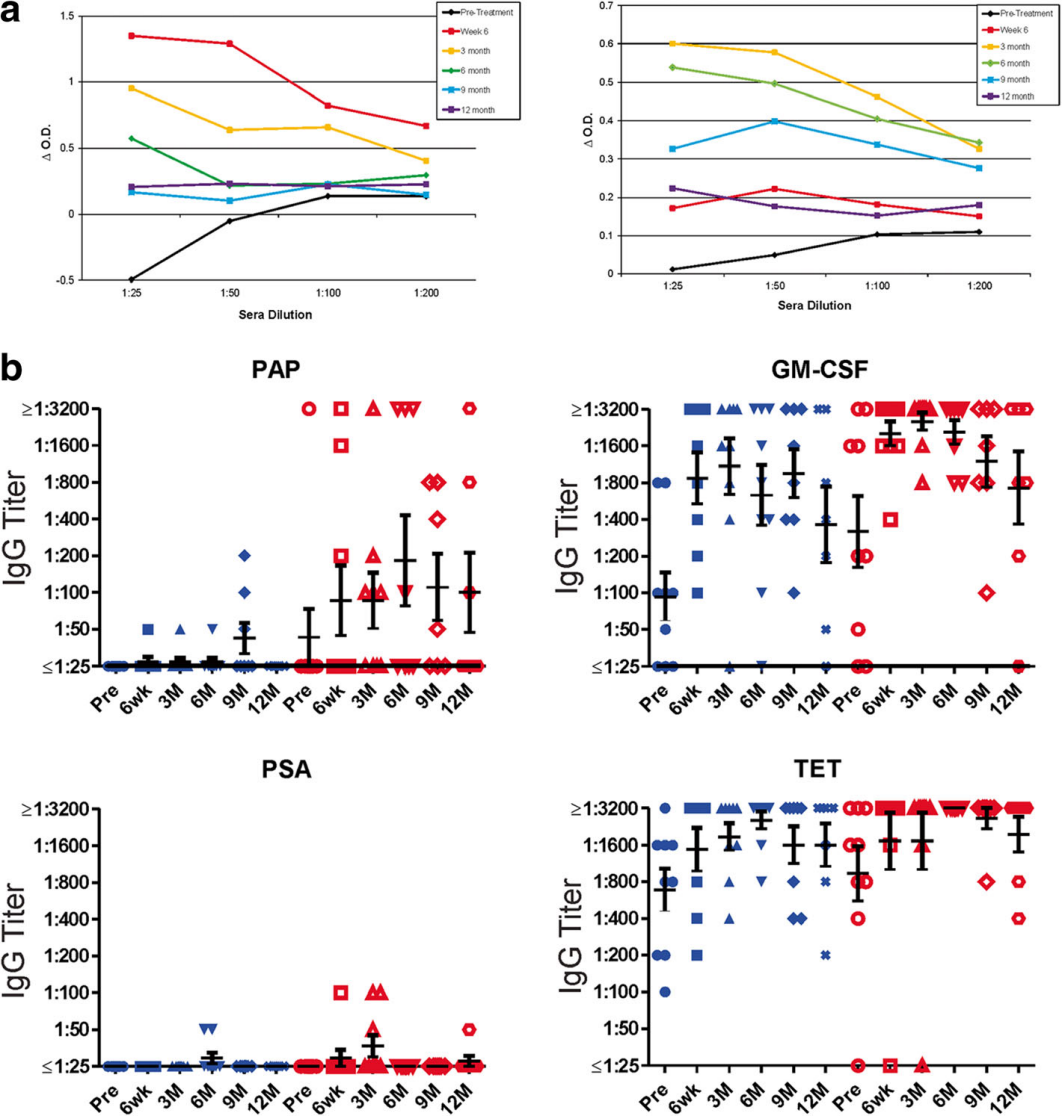
Sipuleucel-T and DNA immunization elicit PAP-specific antibody responses. **a** Shown are results for indirect ELISA evaluating IgG responses to PAP for two individuals (ID010 (right) and ID015 (left)) at each time point at different sera dilutions. ΔO.D. represents the difference in optical density at each serum concentration for PAP relative to blank well. **b** Shown are IgG antibody titers to PAP, GM-CSF, PSA, and tetanus toxoid (TET) for all patients at each time point. Blue symbols represent patients treated in Arm 1, red symbols represent patients treated in Arm 2. Bars show the mean and standard error for each group

In two previous trials using pTVG-HP, we have found that immune responses elicited are Th1 biased, and IgG antibody responses to PAP were not elicited [11, 12]. IgG antibody responses to PAP have been detected, however, following treatment with sipuleucel-T [1, 8]. Consequently, we tested for IgG responses to the same antigens described above (PAP, PSA, tetanus toxoid and GM-CSF). As shown in Fig. 3a for two individual patients treated in Arm 2 with both vaccines, IgG responses to PAP were elicited, in one case peaking at week 6 after treatment with sipuleucel-T and then decreasing in titer, and in the other case not detectable until month 3. IgG titers to all test antigens are shown in Fig. 3b. As expected, antibodies were elicited to tetanus in patients treated on both study arms, and antibody responses to PSA were rare. Antibody responses were also elicited to GM-CSF, and were not different with respect to treatment arm. Antibody responses to PAP, however, were of higher titer and higher frequency in patients treated in Arm 2.

**Fig. 4:**
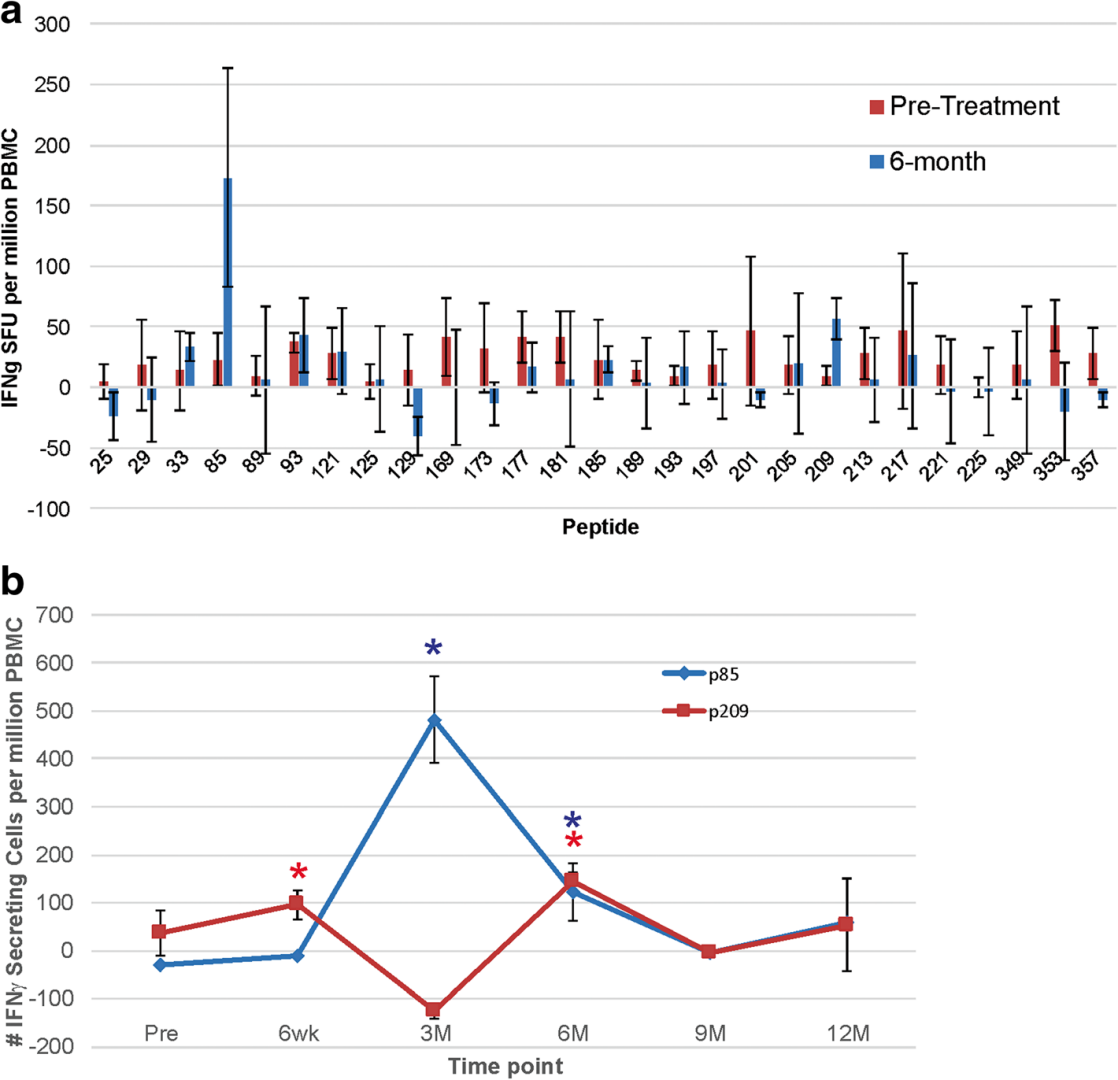
Immune responses to PAP-derived peptides. **a** PBMC from patient ID010 were evaluated at baseline and 6 months for IFNγ ELISPOT response to individual 15-mer peptides derived from the amino acid sequence of PAP. The peptide number indicates the starting amino acid from the PAP sequence of the 15-mer peptide. Shown are mean and standard deviation of IFNγ spot-forming units (SFU) per million PBMC for results, conducted in triplicate for each peptide. **b** PBMC from patient ID010, obtained at the indicated time points, were stimulated in vitro with peptide p85 or p209, and then evaluated for IFNγ release by ELISPOT. Asterisks indicate *p* < 0.05 (t-test) by comparison with media only control

As shown in Fig. 2c, IFNγ-secreting T-cell responses to PAP were detected in only 2 individuals (ID010 and ID017) in Arm 2 (with DNA vaccine). To characterize these responses over time, and to determine if different individual T-cell populations were elicited with sipuleucel-T compared with DNA, samples obtained at 6 months were stimulated in vitro with a panel of 94 15-mer peptides spanning the amino acid sequence of PAP, and then evaluated for individual peptide epitope specificity by ELISPOT. Epitope-specific T cells could not be identified for patient ID017. However, as shown in Fig. 4a, T cells specific for two peptides (p85 and 201) were identified for patient ID010 that were recognized after treatment but not before. Responses to these two peptides were then characterized over time. As shown in Fig. 4b, T cells specific for peptide 209 were detectable as early as 6 weeks, but T cells specific for peptide 85 were not detectable until 3 months. The timing of these responses suggests that sipuleucel-T and DNA vaccination elicited T cells specific for different epitopes, with T cells specific for an epitope contained within p209 elicited with sipuleucel-T, but another in p85 augmented with DNA vaccination.

**Fig. 5:**
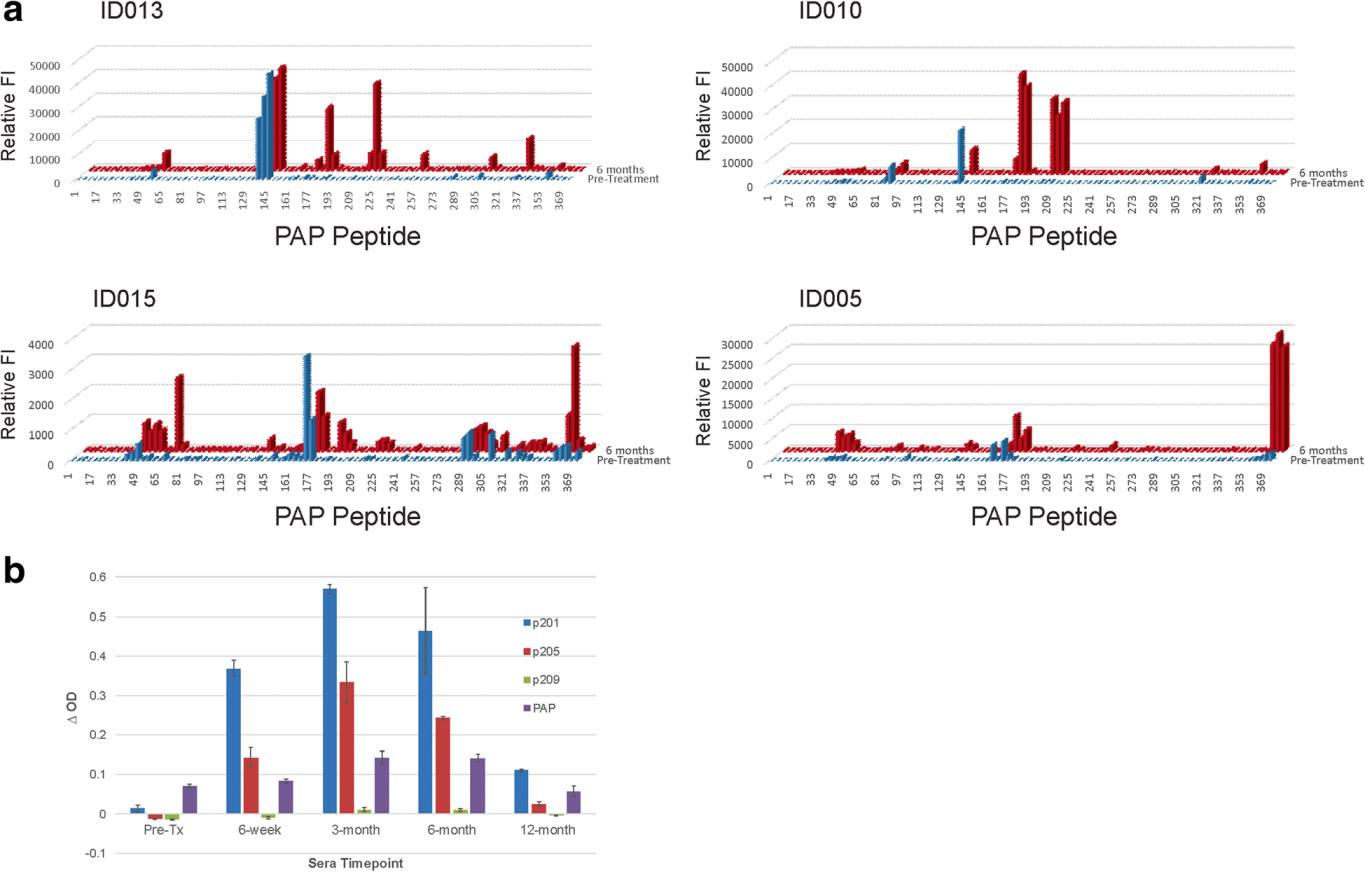
IgG antibodies elicited recognize different epitopes in different patients. **a** 16-mer peptides spanning the amino sequence of PAP were evaluated for IgG antibody response by high-throughput immunoblot. Shown is the relative reactivity (FI, fluorescence intensity) to individual peptides using sera obtained pre-treatment (blue) or at 6 months (red) to each peptide for four separate patients treated in Arm 2. **b** ELISA was used to detect IgG to each of 3 15-mer peptides (beginning at amino acids 201, 205, or 209) or to the full-length PAP protein at the indicated time points. Shown are the mean and standard deviation of triplicate measurements of the Δ O.D. made using sera from patient ID010

To further characterize the antibody responses and determine if IgG responses to B cell epitopes similarly changed over time, antibody responses for four individuals from Arm 2 with the highest titer antibodies were evaluated to a panel of overlapping 16-mer peptides spanning the amino acid sequence of PAP. As demonstrated in Fig. 5a, IgG responses were detectable post-treatment that were not detectable pre-treatment, and regions of the protein recognized were not necessarily shared by different individuals. However, three individuals (ID010, ID013 and ID015) did have IgG responses to peptides within the same region (amino acids 177–232), and these were then characterized over time to determine if responses were associated with response to sipuleucel-T or DNA vaccine. As shown in Fig. 5b for patient ID010, IgG responses to the dominant peptides were detectable by ELISA at 6 weeks, and increased in titer by 3 months, suggesting responses were elicited with sipuleucel-T and further augmented with DNA vaccination. Of note, the antibody epitope recognized in this individual (p201–220) overlapped or was immediately proximal to the T cell epitope also elicited by sipuleucel-T (Fig. 4b). Antibody responses in all patients were determined by ELISA to be IgG, and predominantly IgG1 subtype (data not shown).

### Clinical evaluation

**Fig. 6:**
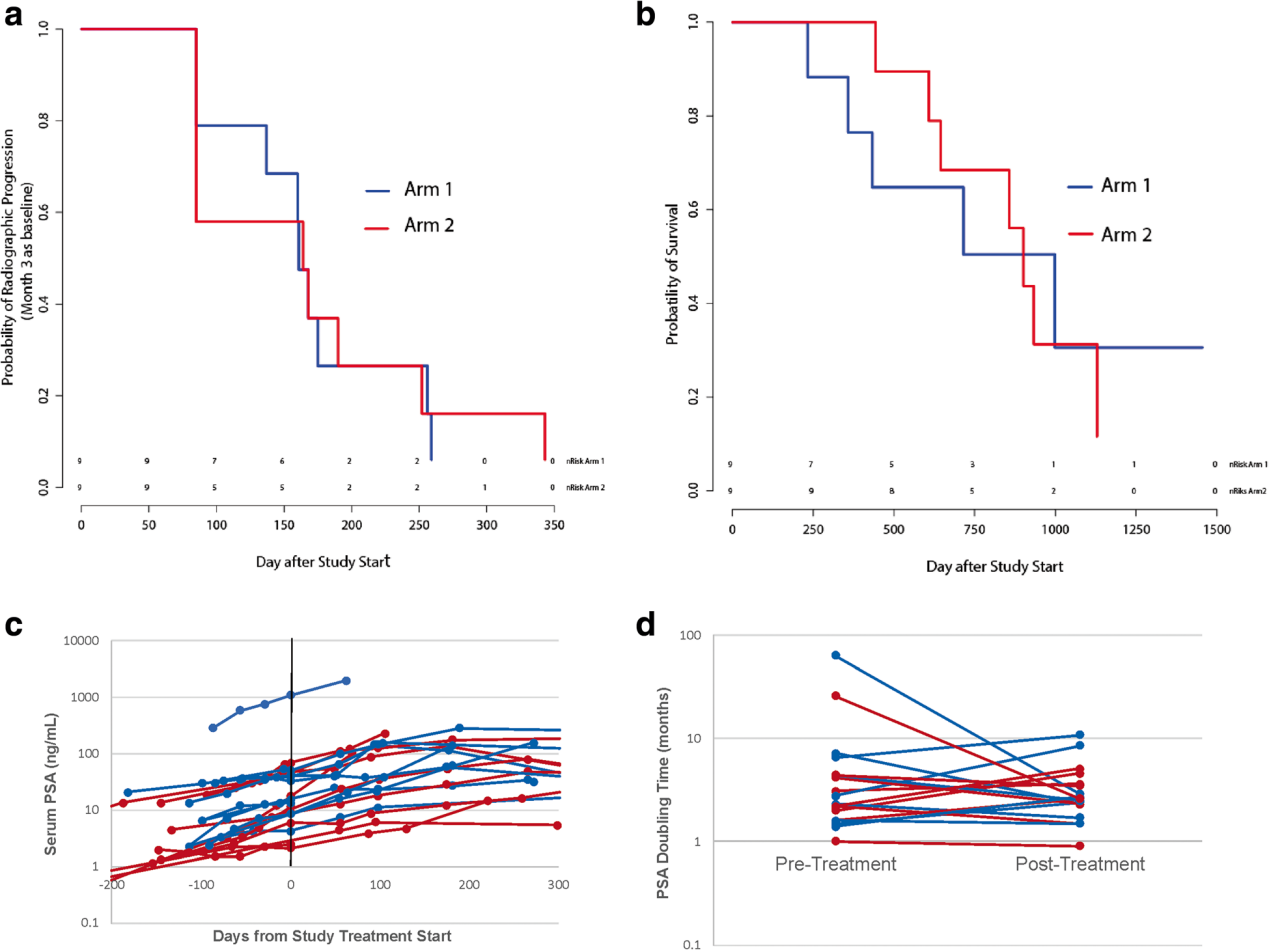
Clinical outcomes. **a** Time to radiographic progression using staging obtained at month 3 as baseline for evaluation. **b** Overall survival. **c** Serum PSA values collected for each patient pre-treatment and up to one year. **d** Pre-treatment and post-treatment PSA doubling times. For each panel, blue lines indicate patients treated in Arm 1, red lines indicate patients treated in Arm 2

In previous placebo-controlled randomized clinical trials conducted with sipuleucel-T, there was not a significant difference in median time to radiographic progression, which was approximately 12 weeks [1, 18]. In order to account for this and possible delayed effects of treatment on progression, patients were evaluated for time to progression using scans obtained at month 3 as a new baseline scan. While the trial was not powered to detect difference in time to progression, as shown in Fig. 6a, there were no differences between study arms in terms of time to progression (median 161 days in Arm 1 vs. 164 days in Arm 2). Of note, however, two patients treated in Arm 2 (ID010 and ID015) had evidence of progression at month 3 compared to baseline, however then remained on trial without progression until month 9 and 12, respectively, at which point radiographic progression beyond month 3 was detected. All other patients with evidence of progression at month 3 had evidence of further progression by month 6. Similarly, while the trial was not powered to detect differences in overall survival, no significant differences in overall survival between study arms were noted (Fig. 6b). Median overall survival was 715 days (24 months) in Arm 1 versus 901 (30 months) in Arm 2 (with DNA vaccine). As shown in Fig. 6c there were no differences observed in PSA kinetics with respect to study arm. PSA doubling times were calculated from PSA values obtained up to 6 months prior to treatment and up to 6 months from day 1 of study treatment. Pre-treatment PSA doubling time was 2.55 months for all patients (2.8 months Arm 1, 2.2 months Arm 2). There were no significant changes in PSA doubling time observed post-treatment (2.6 months overall, 2.5 months Arm 1, 2.6 months Arm 2).

## Discussion

Heterologous prime-boost immunization strategies, in which two different vaccine types are used, each encoding the same antigen, have been demonstrated in many contexts to improve the immunological outcome of vaccination. Studies in preclinical models and human trials have shown increased antigen-specific T cells and/or antibodies using this approach in infectious disease and tumor systems [19–21]. Notably, in the case of viral or bacterial vector vaccines, the use of heterologous prime-boost approaches has been critical to avoid neutralizing immunity to the vector while augmenting immunity to the intended target antigen. This, in fact, was the approach used in the PSA-TRICOM vaccine targeting PSA as a prostate tumor antigen, using vaccinia virus encoding PSA as a priming immunization followed by booster immunizations with fowlpox encoding PSA [22]. We have previously investigated a vaccinia vector encoding PAP and found that multiple immunization with that vector elicited a dominant response to the vector, not the target antigen, and this could be circumvented by booster immunization with either PAP protein or DNA encoding PAP [10]. Given that DNA encoding PAP alone could elicit Th1-biased T cell immunity to PAP without eliciting vector-specific immunity, we have explored it in early clinical trials [11, 12]. At present, the only FDA-approved anti-tumor vaccine is sipuleucel-T, a treatment for advanced prostate cancer that similarly targets the prostate-specific antigen PAP. Given the availability of two vaccines each targeting this tumor antigen, the current trial evaluated whether T-cell responses to PAP could be augmented using them in a prime-boost approach. We found that with this sequence of administration, with sipuleucel-T followed by DNA immunization, there was no evidence of increased Th1-biased response to PAP.

This is the first trial to evaluate long-term effector and memory T cell immunity to PAP following sipuleucel-T treatment. We found that IFNγ- and granzyme B-secreting T cells specific for PAP were amplified with treatment, and could be detected up to at least one year following treatment in some individuals. Of note, PAP-specific granzyme B-secreting immunity was detected in half of patients at week 6 after completing the sipuleucel-T infusions. Antibody and Th1-biased cellular responses were also detected to GM-CSF. This is not surprising, as the PA2024 antigen used for activation of autologous cells in the manufacture of sipuleucel-T is a fusion protein of PAP and GM-CSF. The finding in our study that IFNγ-secreting response specific for GM-CSF were detectable in several individuals (5/16, 31%) who did not have an IFNγ-secreting response specific for PAP likely accounts for previous findings that the frequency of responses to the PA2024 antigen are higher than those detected to the native PAP antigen [1, 8]. That is, many patients likely develop immunity to the GM-CSF portion of the fusion protein. We have previously reported that immunity to GM-CSF can occur following immunization with GM-CSF protein, and to date there has been no evidence of adverse effect from immunity to GM-CSF [23].

This trial was not powered to detect differences in time to progression or overall survival, and no obvious trends were observed. Median overall survival was 28 months, which is consistent with previous trials conducted with sipuleucel-T in this patient population [18]. Embedded within the trial design was an attempt to determine if treatment might slow the progression of disease. Specifically, in previous trials using anti-tumor vaccines conducted in this population, the median progression-free survival was about 12 weeks, at the first radiographic imaging time point [1, 24]. Treatment on the current trial was permitted beyond 12 weeks in order to determine if subsequent imaging showed stable disease, thus our results cannot be directly compared with previous studies using sipuleucel-T in terms of time to progression. Notwithstanding, using this approach, only two patients with evidence of progression at 3 months had stable disease after that, suggesting that delayed disease stability, if it occurs, is not common. Similarly, no differences were observed in pre-treatment and post-treatment PSA doubling times overall or for either treatment arm.

The trial was designed to test whether a DNA vaccine could boost cellular immunity elicited by sipuleucel-T. It was designed in this way given that sipuleucel-T is an approved therapy, and we did not want to potentially delay administration of an approved treatment. In addition, a prescribed course of sipuleucel-T involves three administrations at 2-week intervals, and is not amenable to large schedule interruptions or retreatment at later time points. Thus, the simplest design was to use a DNA vaccine after completing sipuleucel-T treatment. In retrospect, this was likely not the optimal design. First, the majority of patients had disease progression requiring study discontinuation before receiving multiple DNA immunizations, suggesting that, although safe, it may not be clinically feasible to sequence vaccines alone in this patient population when progression occurs quickly. In addition, nearly all studies to date evaluating DNA vaccines in heterologous prime-boost approaches, whether in preclinical models or human trials, have demonstrated that a preferred sequence of immunization is using DNA as the priming immunization [25–30]. In fact, an early study demonstrated that DNA priming followed by a booster immunization with a herpes simplex viral protein elicited a Th1-biased immune response, whereas the opposite sequence elicited a Th2-biased response [31]. Given that sipuleucel-T elicits both Th1 and Th2 immunity to PAP, we suspect this Th2 response was preferentially boosted with DNA leading to an increased IgG response. A preferred approach may have been to use the DNA immunization prior to sipuleucel-T. In preclinical studies using the same DNA vaccine encoding PAP or a *Listeria monocytogenes* vector encoding PAP, we have found that priming with DNA followed by *Listeria* boost, and not the opposite sequence, elicited the most robust Th1-biased cellular immunity and anti-tumor response (manuscript in preparation). Consequently, future studies will explore heterologous prime-boost approaches using DNA as the priming immunization.

## Conclusions

Our findings demonstrate that delivery of two vaccines encoding the same target antigen, using a DNA vaccine as a booster vaccine following treatment with sipuleucel-T, is safe and can augment and diversify the type of immunity elicited with anti-tumor vaccination.
